# Quality-controlled characterization of a monoclonal antibody specific to an EC5-domain of human desmoglein 3 for pemphigus research

**DOI:** 10.3389/fimmu.2024.1464881

**Published:** 2024-10-10

**Authors:** Rüdiger Eming, Shafaq Riaz, Eliane J. Müller, Anna Zakrzewicz, Uwe Linne, Ritva Tikkanen, Christine Lea Zimmer, Christoph Hudemann

**Affiliations:** ^1^ Department of Dermatology and Allergology, Philipps University Marburg, Marburg, Germany; ^2^ Department of Dermatology, Venerology and Allergology, German Armed Forces Central Hospital Koblenz, Koblenz, Germany; ^3^ Department for BioMedical Research, Molecular Dermatology and Stem Cell Research, University of Bern, Bern, Switzerland; ^4^ Department of Dermatology, Inselspital, Bern University Hospital, University of Bern, Bern, Switzerland; ^5^ Institute of Biochemistry, Medical Faculty, Justus-Liebig-University Giessen, Giessen, Germany; ^6^ Mass Spectrometry Facility, Department of Chemistry, Philipps University, Marburg, Germany

**Keywords:** quality control, antibody, pemphigus vulgaris, autoimmunity, desmoglein (Dsg), 2G4, PV

## Abstract

**Background:**

Pemphigus vulgaris (PV) is a life-threatening autoimmune blistering disease caused mainly by IgG autoantibodies (auto-abs) against the cadherin-type adhesion molecules desmoglein (Dsg) 1 and 3. Pathogenic anti-Dsg3 auto-abs bind to different Dsg3 epitopes, leading, among others, to signalling that is involved in pathogenic events, such as Dsg3 depletion. As central tools in research on PV, a limited number of antibodies such as AK23 are frequently used by the autoimmune bullous disease community.

**Methods:**

Previously, we have introduced a novel Dsg3 EC5-binding antibody termed 2G4 that may potentially serve as a superior tool for numerous PV related analysis. The purpose of this study was to develop a quality-controlled production and verification process that allows I) a continuous quality improvement, and II) a verified and comprehensible overall quality with regard to pathogenic antigen-specific binding in a variety of pemphigus assays for each batch production.

**Results:**

Thus, a workflow based on a standardized operating procedure was established. This includes the verification of purity and *in-vitro* binding capacity (SDS-page, direct and indirect immunofluorescence) as primary parameters, and size analysis by mass-spectrometry and *ex-vivo* pathogenicity by monolayer dissociation assay.

**Conclusion:**

We here present an extensive point-by-point quality controlled IgG production protocol, which will serve as a basis for a standardized antibody assessment in PV research.

## Graphical Abstract

Streamline process of controlled 2G4 IgG production and verification. Straight lines display compulsory verification parameters, while dashed lines display optional analysis. SOP, standard operating procedures; IF, immunofluorescence. Created with BioRender.com.

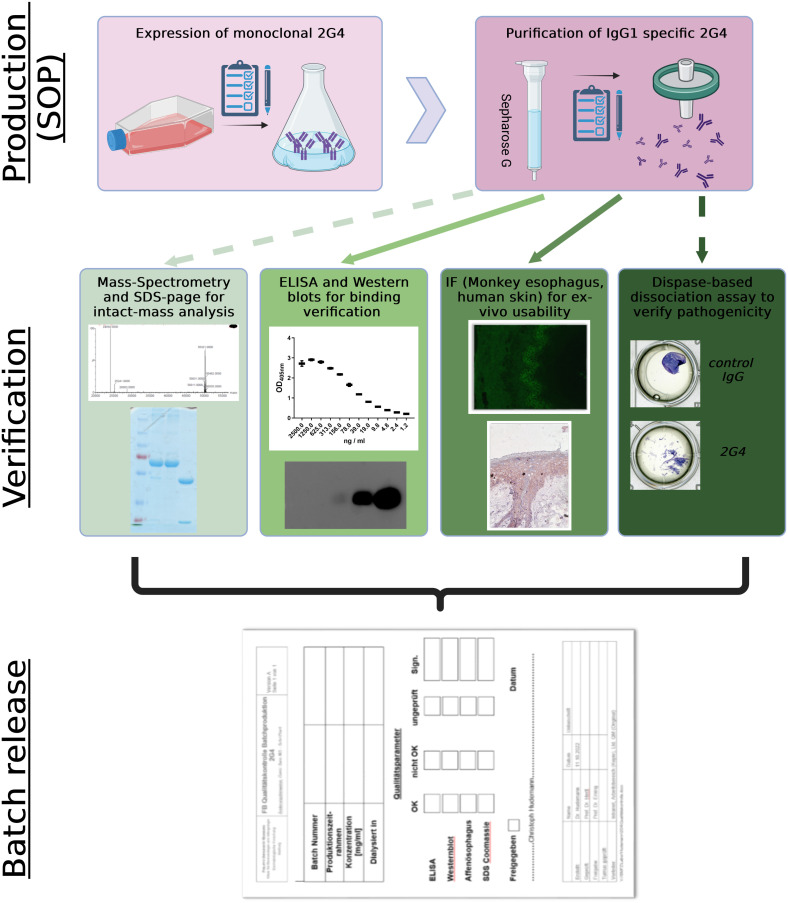

## Introduction

Pemphigus is a potentially lethal IgG autoantibody (auto-ab) - driven autoimmune disorder affecting mucous membranes and the skin (1). The auto-ab response in pemphigus is polyclonal. While immunoglobulin 4 (IgG4) abs are predominantly found in the sera of patients with an active pemphigus disease, antigen-specific IgG1 frequently associates with the initiation or remittent stage, together with IgG2 and IgG3 (2). In monoclonal and pathogenic antibodies, switching IgG1 and IgG4 subclasses does not directly affect their antigen binding or pathogenic properties (3).

Pemphigus can be divided in two major subtypes, depending on the involved auto-ab antigen profile. Characteristic for pemphigus foliaceus are desmoglein 1 (Dsg1)-specific auto-ab that induce sub-corneal blister formation in the epidermis (4). In pemphigus vulgaris (PV) however, mainly Dsg3-specific auto-abs induce acantholysis in the basal and supra-basal layers of the mucous membranes, resulting in painful and slow-healing sores (5). Desmogleins belong to the group of desmosomal cadherins, and their main functions confers to epidermal keratinocyte adhesion. In humans, separate genes encode four desmogleins (Dsg1-Dsg4). They comprise five extracellular cadherin domains (EC1-EC5), a single-pass transmembrane domain (TMD), and an intracellular domain that associates with desmosomal plaque proteins (6, 7). The EC1–EC3 domains of Dsg3 are highly homologous to the EC1–3 of Dsg1 (75–80% identity) (8). Several studies have shown that most pemphigus-specific auto-ab specifically bind to the N-terminal EC1 domain of Dsg3 that also functions as the major mediator of homo- and heterophilic interactions (9). Overall, Dsg3-specific IgG reactivity correlates with disease activity, and approximately 80% of PV patients exhibit serum IgG directed against the EC1-2 domains, followed by EC3 (15%), EC4 (21%) and EC5 (17%) (10).

The desmoglein compensation theory, initially proposed by Mahoney, Amagai and Stanley, postulates that Dsg3 compensates for the loss of Dsg1 in the mucous membrane, only leading to clinically active erosions in the skin. In contrast, anti-Dsg3 IgG leads to an impairment of mucosal epidermal adhesion due to the low expression of Dsg1 in this tissue, which cannot fully compensate the loss of Dsg3 adhesion (4). Although the Dsg compensation theory well reflects the clinical features of pemphigus, a growing pool of recent studies suggests a more diverse antigen-specific picture that potentially contributes to individual antibody pathogenesis (11). This underlines the need for a higher degree of personalized medicine that takes the auto-ab profiles into account when planning treatment strategies. Additionally, the interplay between pathogenic and non-pathogenic desmoglein-specific IgG may potentially add a synergistic effect by, for instance, causing a p38-dependent antigen-clustering. While most anti-EC1 or -EC2 abs are directly contributing to the clinical phenotype due to their pathogenicity, those targeting EC3–5 are mainly considered as ‘synergistic and semipathogenic’ autoantibodies (12). For *in-vitro* and *in-vivo* studies, EC1-specific pathogenic antibodies, such as the widely distributed murine monoclonal IgG antibody AK23, are typically used (13). In a study by Hudemann et al. (14), a novel EC5-specific anti-Dsg3 ab (2G4) was described. In this study, comprehensive evidence was provided that binding to the Dsg3 EC5 domain leads to loss of epidermal adhesion in human and mouse skin together with exofoliative toxin, challenging the concept that only IgG directed against the EC1 subdomain of Dsg3 is pathogenic. Further mechanistic analysis revealed similar effects on keratin retraction and reduction of desmosome number as with AK23, while only AK23 -mediated effects but not these of 2G4 could be ameliorated by Src inhibition (15).

Quality assurance in a laboratory is of utmost significance in a field of research, such as pemphigus, due to the narrow variety of tools used for *ex-vivo* and *in-vivo* studies. We believe that the standardized distribution of PV samples or antibodies is crucial for reliable and accurate clinical and preclinical results. The aim of this study was to establish and verify coordinated activities to direct and control 2G4 IgG production as a potentially central tool in pemphigus research, allowing comparable data generation of constant quality throughout different laboratory sites and times. We therefore implemented an analysis pipeline, including standard molecular analysis (gel electrophoresis, ELISA, mass spectrometry) followed by routine diagnostic analysis using indirect immunofluorescence on monkey esophagus and human tissue (16) and verification of pathogenicity by monolayer dissociation assay (MDA), in order to ensure a functional product allowing standardized analysis of downstream clinical and preclinical samples. Introduction of such quality control mechanisms will potentially harmonize pemphigus research.

## Materials and methods

### Characterization of hybridoma cells

Purified Dsg3 (in house) was labelled with Alexa Fluor (AF) 647 (Thermo Fischer Scientific, Waltham, USA) or phycoerythrin (PE) (Abcam, Cambridge, UK) according to manufacturer’s instructions, followed by titration for optimal staining concentrations. The 2G4 B cell hybridoma was characterized by flow cytometry using a BD LSR Fortessa equipped with four lasers (BD Biosciences, San Jose, CA, USA) and analyzed in FlowJo Version 10.8 (BD Biosciences, San Jose, CA, USA). Cells were found to be anti-mouse IgG (AF488; Abcam) and CD138 (BV786, Biolegend, San Diego, USA) positive after dead cell exclusion by Zombie NIRTM Fixable Viability Kit (BioLegend, San Diego, CA). Dsg3–specificity was further shown by double-positivity using two similar Dsg3-protein batches labeled with either AF647 or PE (**Supplementary Figure 1**).

### Antibody production and purification

Dsg3(EC5)-specific monoclonal antibody 2G4 was produced as described previously (14). In brief, hybridoma culture supernatants without serum additive were collected after seven days. Supernatant IgG antibodies were purified by affinity chromatography using protein G columns (GE-Healthcare, Munich, Germany) following standard operating procedures. The eluate was collected in a small amount of neutralisation buffer (Tris-HCl, pH 9) and sterile filtered with 0.22 µm filters (LLG Labware, Meckenheim, Germany). Finally, the purified 2G4 was taken up in PBS and 3 mM NaAc pH 7.5 and aliquoted for further characterization. The mouse anti-Dsg3 antibody AK23 was produced according to Zakrzewicz et al. (17).

### Mass spectrometric antibody verification

The solubilized antibody was reduced by adding TCEP (Tris(2-carboxyethyl)phosphine) to a final concentration of 5 mM. After incubation for 60 minutes at room temperature, 2 µL of the buffered protein solution was desalted using a Waters ACQUITY H-Class HPLC-system equipped with a MassPrep column (Waters, MA, USA). Desalted proteins were eluted into the ESI source of a Synapt G2Si mass spectrometer (Waters) using a gradient of buffer A (water/0.05% formic acid) and buffer B (acetonitrile/0.045% formic acid) at a column temperature of 60°C and a flow rate of 0.1 mL/minutes: Isocratic elution with 5% A for two minutes, followed by a linear gradient to 95% B within 8 minutes and holding 95% B for additional 4 minutes.

Positive ions within the mass range of 500-5000 m/z were detected. Glu-Fibrinopeptide B was measured every 45 s for automatic mass drift correction. Averaged spectra were deconvoluted after baseline subtraction and eventual smoothing using MassLynx instrument software with MaxEnt1 extension.

### Enzyme linked immunosorbent assay

ELISA was performed as previously described (10). Briefly, the extracellular domain of human Dsg3 was produced in baculovirus-infected insect cells (High Five; Invitrogen, Carlsbad, CA, USA) as described previously (18). Coating onto immunomicrotitre plate (96-Well; Greiner Bio-one, Frickenhausen, Germany) was done for 24 h at a concentration of 5 µg/ml. As primary antibodies, different batches of 2G4 (2.5 µg/ml or less) were applied. For detection, species-specific HRP-conjugated secondary antibodies (1:2000, Dako, Glostrup, Denmark) were used. Absorbance level was measured at 405 nm (Tecan plate reader Sunrise + Magellan software; Tecan Group Ltd., Männedorf, Switzerland).

### SDS-page and western blot analysis

Coomassie staining of SDS-page run with freshly purified 2G4 was performed using a standard protocol. Purity analysis was defined using ImageJ (V. 1.52a, Rasband, USA). The integration of the Area Under the Curve (AUC) was calculated for all the proteins (representing 100%) over the area for the antibody light (25 kDa) and heavy chains (50 kDa) (purity coefficient >0.8). 2G4 antibody-specificity was then analysed by western blot according to standard procedures. Horseradish peroxidase (HRP)-conjugated anti-mouse IgG (1:2000; Dako, Glostrup, Denmark) served as secondary antibodies. Antibody binding was visualized by a commercial HRP substrate (Immobilon Western Chemiluminescent HRP substrate; Millipore, Billerica, MA). Signals were detected by a digital chemiluminescence reader (PEQLAB, Erlangen, Germany).

### Immunohistochemical analysis

Formalin fixed tissue where embedded in paraffin, and 3 µm thick paraffin sections were counter-stained with hematoxylin and eosin (HE). The HE-stained sections were microscopically assessed and random images collected under the 10 × objective (Keyence, Osaka, JP). For histological analysis, sections were rehydrated twice for 5 minutes with PBS, treated with 3% H_2_O_2_ for 10 minutes to quench endogenous peroxidase activity, and washed twice with PBS. Non-specific binding was blocked with 10% fetal calf serum (FCS) for 1 h. Slides were incubated overnight at 4°C or for 1 h at room temperature with 2G4 IgG diluted 1:100. The primary antibody was omitted for negative control. After three 5 minutes washes in PBS, the slides were incubated with anti-mouse IgG-HRP (Dako, Glostrup, Denmark), diluted 1:2000, for 1 h at room temperature. Following three additional washing steps, the sections were incubated with the substrate aminoethyl carbazole (AEC, Invitrogen, Karlsruhe, Germany) for 5 min at room temperature, counter-stained with Mayer’s hematoxylin and mounted with solvent-free medium Fluoromount with DAPI (Thermo Fischer Scientific, Darmstadt, Germany).

### Indirect antibody immunofluorescence

Monkey oesophagus (ME) sections were obtained from SCIMEDX (Dover, NJ, USA). The slides were blocked with TBS-Ca^2+^/1% BSA at room temperature (RT) for 30 minutes, then washed with TBS-Ca^2+^ three times and incubated with serum samples diluted 1:1000 – 1:10000 (and, additionally, 1:10 in case of negative immunofluorescence results at 1:100) in TBS-Ca^2+^/1% BSA at RT for 60 min. The bound antibodies were detected with FITC-conjugated anti-murine IgG F(ab’)_2_ (dilution 1:100; Bio-Rad, CA, USA). Finally, the slides were washed in PBS for 15 minutes and mounted with DAPI Fluoromount-G mounting medium (SouthernBiotech, Alabama, USA).

### Monolayer dissociation assay

The human keratinocyte cell line hTert/KER-CT (ATCC^®^, CRL4048) was seeded in a 24 well plate. At confluency, KGM2 (Promocell, Heidelberg, Germany) (0.05 mM CaCl_2_) was exchanged to KGM2 with 2 mM CaCl_2_ for 24 h. Cells were then treated with either AK23 (75 µg/mL), 2G4 (75 µg/mL) or human control-IgG (75 µg/mL) in triplicates for 24 h at 37°C and 5% CO_2_. The cells were washed with HBSS (Gibco, Karlsruhe, Germany) and incubated with 2.5 U/mL Dispase II (Roche, Mannheim, Germany) until the monolayers were completely detached. Subsequently, the cells were incubated with 4,5-dimethylthiazol-2-yl)-2,5-diphenyltetrazolium bromide (MTT) (0.25 mg/mL) for better visualization for 15 minutes. For fragmentation, mechanical stress was applied to the monolayer by pipetting up and down with a 1ml pipette (19). The same conditions were used for all samples. The fragments were fixed using 4% paraformaldehyde and counted automatically using the ImageJ software.

### Statistical analysis

Statistical analysis was performed using GraphPad Prism 6.02 (GraphPad Software Inc., La Jolla, USA). Cumulative data are displayed as box plots with median. For group comparisons, two-tailed nonparametric ANOVA multiple comparison analysis (ELISA) or a non-parametric Kruskal-Wallis multiple comparison (between AK23 and batches) was performed (MDA assay). Differences between the groups were considered as statistically significant at *p* values of <0.05.

## Results

**Figure 1:**
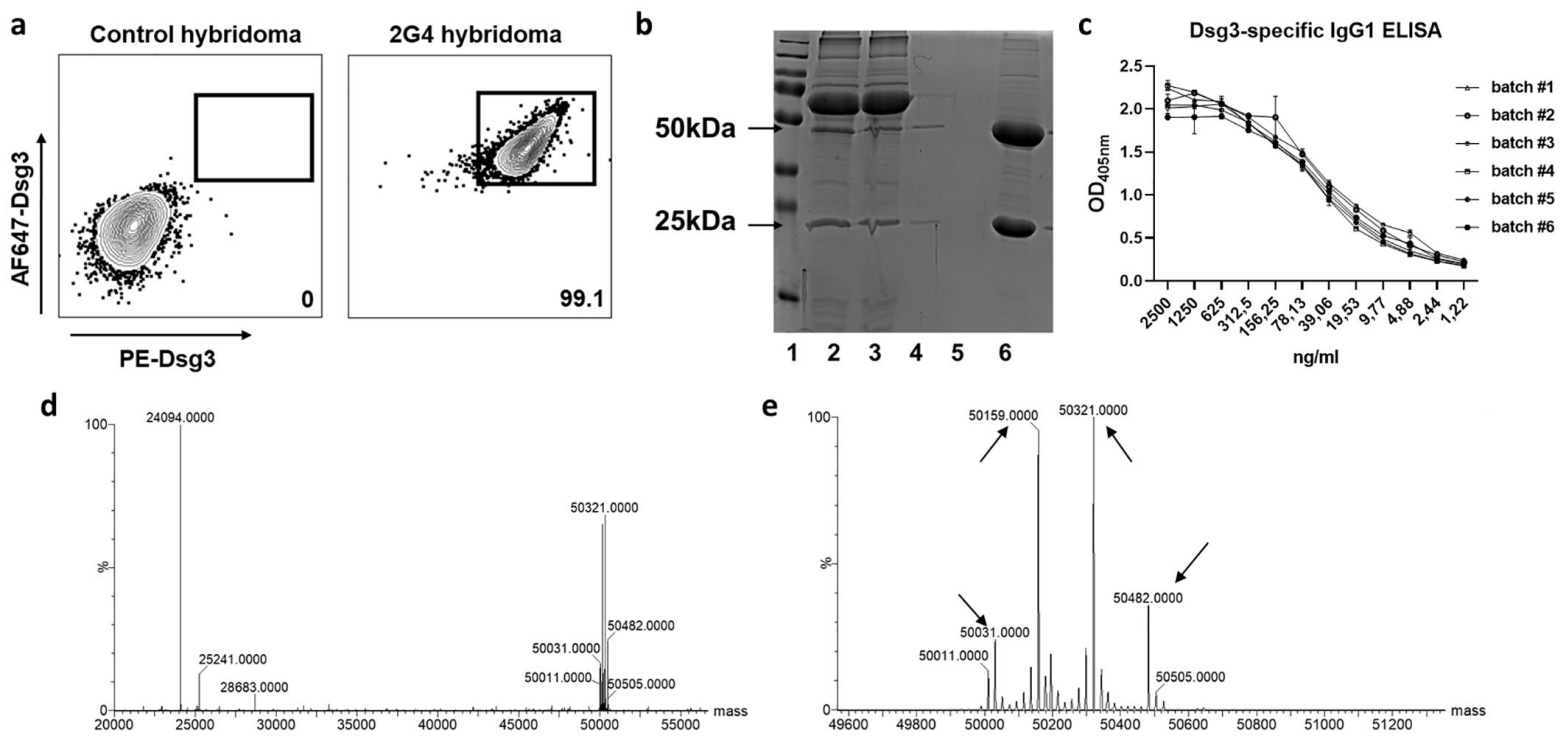
Hybridoma characterization and antibody size and structural verification. **(A)** Representative flow cytometry of the 2G4 hybridoma cell line. Cells were gated as life singlet CD138+ IgG+, and Dsg3–specific cells were identified as dual stained for Dsg3-AF647 and Dsg3-PE. **(B)** Exemplified reducing SDS-page for purity analysis. Lane 1=marker, 2/3=medium before and after agarose binding, 4/5=flow trough, 6=elution (>90% IgG purity). **(C)** Dsg3 enzyme-linked immunosorbent assay applying a dilution series of 2G4 batches #1-6 (optical density [OD] at 405 nm). **(D)** Mass spectrometric analysis confirms the presence of a monoclonal antibody. Reduction with dithiothreitol leads to separation of heavy and light chain. **(E)** Zoom into the heavy chain reveals 4 glycosylation sites indicated by arrows.

The implemented standard operation procedures contain a three-step quality control consisting of the actual production, verification analysis and, if all parameters are successfully passed, the batch release. We further compared variations between different batches of the monoclonal 2G4 anti-Dsg3 IgG produced over a time period of one year (**Table 1**) to verify consistent functionality. Dsg3-specificity of the hybridoma line used was verified by FACS via initial gating on CD138 (plasma cells) and IgG positivity (**Supplementary Figure 1**). To avoid fluorochrome-based identification of false positive cells, Dsg3 was fluorescently labelled with either PE or AF647, and double-positive cells were identified with a 99.1% reactivity compared to an unrelated hybridoma cell line (**Figure 1A**). After purification, initial IgG purity was analysed via SDS-page quantification, whereupon the standard purity lies above 91% (25 kDa light –and 50 kDa heavy –antibody chain/unspecific bands) (exemplified in **Figure 1B**). Dsg3-specific IgG1 ELISA revealed a high sensitivity against hDsg3, with comparable standard curves over a variety of batches (**Figure 1C**) without major outliers (**Supplementary Table 1**). After reduction with TCEP, light (23742 m/z) and heavy (main signal: 49858 m/z) chains of the antibody were measured by intact protein mass spectrometry. Defined signals for light and heavy chain indicated that the antibody was of monoclonal origin (**Figure 1D**). Furthermore, for the heavy chain, two additional signals could be detected (49696 m/z and 50020 m/z) with a mass difference of 162 Da compared to the main signal (**Figure 1E**). This suggested heterogenous heavy chains glycosylation with different glycan variants.

**Table 1:** 2G4 batch characteristics.

Batch #	Purification date	Concentration [mg/ml]	Final amount [mg]
1 - 02/2022	18.02.2022	1,19	1,19
2 - 03/2022	31.05.2022	1,14	5
3 - 04/2022	15.08.2022	0,98	4,51
4 - 06/2022	05.04.2022	1,13	2,7
5 - 07/2022	19.04.2022	0,95	4,18
6 - 08/2022	26.10.2022	1,11	3,9

**Figure 2:**
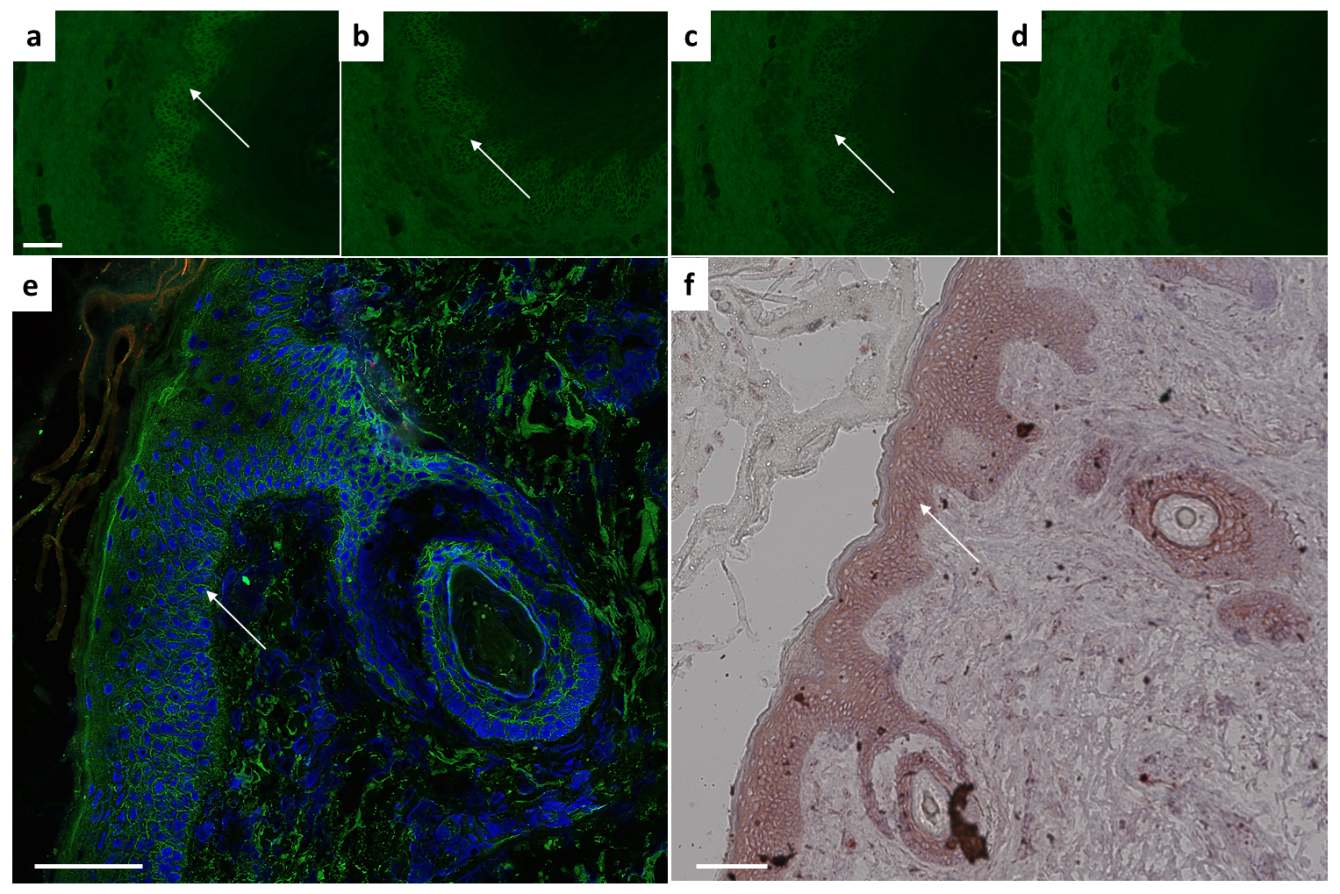
*Ex vivo* binding verification using monkey esophagus or human skin sections. Characteristic intercellular epithelial staining on monkey esophagus visible with 2G4 as primary antibody at dilutions of **(A)** 1:1000, **(B)** 1:5000, **(C)** 1:10.000 indicated by arrow. **(D)** Control sample remains negative. Dsg3 characteristic basal and immediate suprabasal layers of skin visible in human skin on **(E)** cryosections (IgG green, DAPI blue) or **(F)** paraffin-embedded sections. Scale bar = 100µm.

Following structural integrity analysis, *in-vitro* binding ability was assessed by indirect immunofluorescence on monkey oesophagus. Different dilutions up to 1:10.000 revealed the presence of desmosome-binding IgG (**Figures 2A–D**). To ensure antibody validity, we next performed histological analysis on fixed cryosections (by immunofluorescence, IF) and paraffin-embedded human skin samples (by chromogenic staining). In the adult human and mouse skin, Dsg3 distribution is primarily restricted to the basal and immediate suprabasal cell layers (20, 21). Here, we could verify a clear basal and immediate subrabasal membrane staining in human epidermis and hair follicles (**Figure 2E**). Paraffin-embedded histological skin sections revealed an epithelial intermembrane staining pattern. The expected basal and suprabasal distribution separation, however, was less pronounced compared to IF (**Figure 2F**).

**Figure 3:**
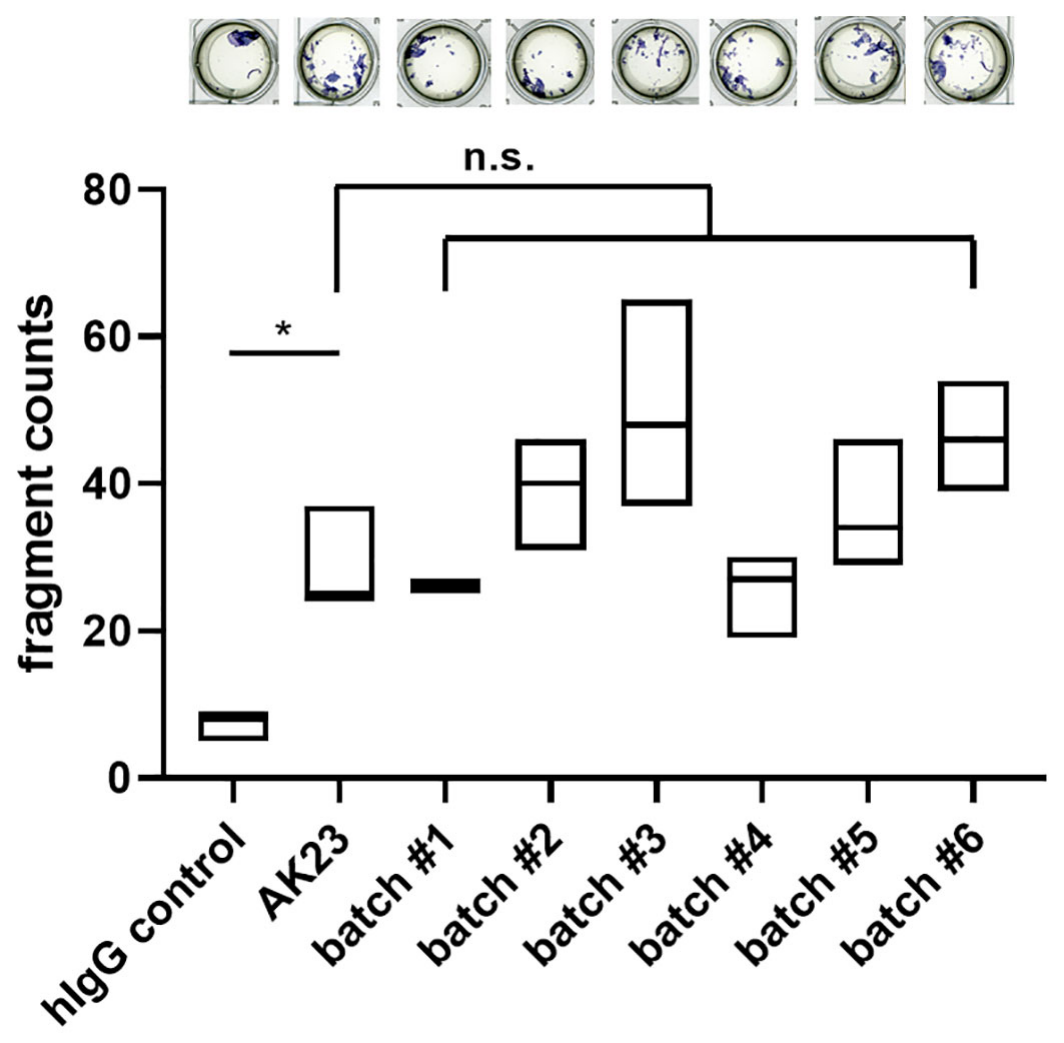
Batch dependent 2G4 analysis confirms similar pathogenicity based upon disruption of desmosomes. Monolayer dissociation assay using human hTert-immortalized keratinocytes treated with 2G4, AK23, or human control IgG (all at 75 µg/ml for 24 h), n=triplicates/batch. *P <0.05, ns not significant.

For the analysis of pathogenicity, a batch comparison was performed using the MDA with human hTert-immortalized keratinocytes. In comparison to hIgG control treatment, application of AK23 induced a significant increase in the fragmentation. All 2G4 batches induced similar fragment counts compared to AK23 (**Figure 3**). In conclusion, we here present an antibody validation procedure ensuring similar quality in terms of structural and functional aspects.

## Discussion

Pemphigus is a rare autoimmune blistering disease, mediated by a heterogeneous mixture of both pathogenic and non-pathogenic serum IgG mainly directed against the desmosomal adhesion protein Dsg3, a key player in maintaining epidermal integrity. Large scale mapping studies of PV sera showed that 91% of PV sera mapped to the Dsg3 N-terminal domains EC1-2, which are crucial for cis- and trans-adhesive interactions of the desmogleins (22). Non-pathogenic or C-terminal anti-Dsg3 IgG may contribute indirectly via epitope spreading to the pathogenicity of the polyclonal anti-Dsg3 IgG pool (23). Therefore, the “multiple hit theory” has been postulated to express the interplay of a variety of antibodies as a prerequisite to induce pemphigus (24). Extrapolation of the inherent patient specific IgG heterogeneity *ex-vivo* and *in-vitro* has been a widely discussed field, raising the need for novel pathogenic and non-pathogenic PV-specific antibodies with different antigen-specific affinities. In this study, we aimed at developing a quality controlled production pipeline for the first anti-EC5 human Dsg3-specific, murine monoclonal antibody (2G4) that originates from a well characterized B cell hybridoma (14), in order to ensure similar structural and functional properties of the purified antibody (see graphical abstract). Ultimately, we strived to ensure equal quality of 2G4 either as a positive control in analytical assays or as a validated basic tool for *ex-vivo* analysis.

Studies of antigen-specific cells in an autoimmune context are challenging due to the low prevalence, and lack of methods for their accurate identification. To reduce the background attributed to the fluorochrome – antibody binding itself, the use of dual antigen-specific labelling by two fluorochromes for cell selection has been suggested (25). This targeting approach has now been widely used for the identification of antigen-specific B cells, also in pemphigus (26, 27). Here, we could show an expected ≥99% positivity for the 2G4 hybridoma B cells using Dsg3 with both AF647 and PE as fluorochromes (**Figure 1A**). In addition to patient samples, this can potentially serve as a potent tool to unravel Dsg3-specific B cell functionality in preclinical PV mouse models using IHC and other methods. (28), as described in (29).

As shown in (14), the epitope of 2G4 lies within the extracellular EC5 domain of human desmoglein 3, contrasting the EC1/EC2 specificity of AK23. By using reducing SDS-Page we determined the antibody purity, and eventual aggregation or degradation. Extensive aggregation can lead to antibody precipitation, potentially affecting biological activity, and may also increase immunogenic responses (30). We found a consistent purity of ≥ 91%, and additional downstream protein liquid chromatography could potentially increase the purity even further. Cell-culture quality liquids were used throughout the entire process, while mycoplasma-negativity is routinely monitored. Whether endotoxin-presence potentially affects any of the parameters (31) was not further assessed. Additionally, we used intact protein mass spectrometry to validate the molecular integrity of the antibody and its glycosylation signature. In general, intact protein mass spectrometry reveals amino acid exchanges, length variations of the polypeptide chains, or changes in the glycosylation patterns. Upon measuring the antibody under denaturing conditions, we detected distinct signals for the light and heavy chains, confirming the monoclonal nature of the antibody. Furthermore, as expected, glycosylation variants were present for the heavy chain (mass difference 162 Da each) (**Figure 1E**).

The final antibody validation step for any application is to demonstrate intra- and inter-assay - reproducibility. By performing batch-comparisons using the most common applications, ELISA and MDA, we could not only show similar batch quality in the ELISA (**Figure 1C**), but also equal functionality by comparable pathogenicity towards the most commonly used monoclonal pemphigus antibody AK23 (**Figure 3**). Differential IgG glycosylation is a powerful post-translational modification that was found to affect several autoimmune diseases by modifying receptor-mediated effector functions and half-life (32). While pemphigus antigens Dsg1 and Dsg3 are functionally glycosylation independent (33), the glycosylation profile of PV IgG is drastically altered (34). It is therefore of interest to further evaluate the glycosylation profile of 2G4 after production (see **Figure 1D**), but this would be beyond the scope of this study.

In this report, we have introduced a quality control approach to assure constant quality and functionality of the 2G4 antibody reflecting, at least in parts, the pathogenicity of PV IgG. While additional analytical techniques such as differential scanning fluorometry could provide deeper insights in the nature of antibody affinity (35), this analysis would certainly exceed its means as standard technique. Using a murine monoclonal antibody certainly reflects but a part of the pathogenicity of a pool of pemphigus IgG. However, having established a quality controlled production pipeline of one parameter (EC5-specific pathogenicity) provides a consistent standard eliminating batch variations. Quality controlled 2G4 antibody could thus, ideally in combination with other widely used antibodies such as, for example, the EC1/2 specific AK23, reflect a heterogenic pemphigus specific IgG pool to ultimately booster pemphigus research.

## Data Availability

The original contributions presented in the study are included in the article/**Supplementary Material**. Further inquiries can be directed to the corresponding author.
