# Supplementary material for: Quality-controlled characterization of a monoclonal antibody specific to an EC5-domain of human desmoglein 3 for pemphigus research
Source: Front Immunol. 2024 Oct 10;15:1464881. doi: 10.3389/fimmu.2024.1464881 (PMC11499099; doi:10.3389/fimmu.2024.1464881)
Supplement: Supplementary file 1 [file DataSheet1.docx]

**Quality-controlled characterization of an EC5 specific monoclonal antibody against Desmoglein 3 as a standardized tool for pemphigus analysis**

- **Supplementary -**

Eming R.^1,2^, Riaz S.^1^, Müller E.J.^3^, Zakrzewicz A.^4^, Linne U.^5^, Tikkanen R. ^4^, Zimmer CL.^1^, Hudemann C. ^1, *^

^1^ Department of Dermatology and Allergology, Philipps University Marburg, Marburg, Germany

^2^ Department of Dermatology, Venerology and Allergology, German Armed Forces Central Hospital Koblenz, 56072 Koblenz, Germany.

^3^ Department for BioMedical Research, Molecular Dermatology and Stem Cell Research, University of Bern, Switzerland.

^4^ Institute of Biochemistry, Medical Faculty, Justus-Liebig-University Giessen, Giessen, Germany.

^5^ Mass Spectrometry Facility, Department of Chemistry, Philipps University, Marburg, Germany

Figures: 1

Tables: 1

*Keywords:* quality control, antibody, pemphigus vulgaris, autoimmunity

**Supplementary tables**

|  | **2G4**  **2500ng/ml** | **2G4**  **1250ng/ml** | **2G4**  **625ng/ml** | **2G4**  **312,5ng/ml** | **2G4**  **156,2ng/ml** | **2G4**  **78,13ng/ml** | **2G4**  **39,06ng/ml** | **2G4**  **19,53ng/ml** | **2G4**  **9,77ng/ml** | **2G4**  **4,88ng/ml** | **2G4**  **2,44ng/ml** | **2G4**  **1,22ng/ml** |
| --- | --- | --- | --- | --- | --- | --- | --- | --- | --- | --- | --- | --- |
| **batch #1 vs. batch #2** | 0,2859 | 0,8576 | 0,9986 | 0,7327 | **0,001** | 0,4358 | 0,7327 | 0,4823 | 0,6502 | 0,9397 | 0,9768 | 0,9983 |
| **batch #1 vs. batch #3** | **0,0139** | 0,8542 | 0,9977 | 0,8705 | 0,9619 | 0,237 | 0,3911 | 0,1452 | 0,1341 | **0,0311** | 0,897 | 0,9788 |
| **batch #1 vs. batch #4** | 0,9966 | 0,7754 | 0,9973 | >0,9999 | 0,9942 | >0,9999 | 0,9377 | 0,6596 | 0,9619 | 0,9889 | 0,9993 | 0,9996 |
| **batch #1 vs. batch #5** | 0,0548 | 0,9416 | 0,6502 | >0,9999 | 0,97 | >0,9999 | 0,9724 | 0,9983 | 0,9969 | 0,9969 | 0,9996 | >0,9999 |
| **batch #1 vs. batch #6** | **<0,0001** | **0,0436** | 0,1119 | 0,8608 | >0,9999 | 0,9938 | 0,9934 | 0,9983 | 0,9869 | 0,7995 | >0,9999 | >0,9999 |
| **batch #2 vs. batch #3** | 0,8034 | 0,2028 | >0,9999 | 0,9998 | **0,0142** | 0,999 | 0,9942 | 0,9824 | 0,9201 | 0,2572 | 0,9995 | 0,9996 |
| **batch #2 vs. batch #4** | 0,1082 | >0,9999 | >0,9999 | 0,7836 | **0,0001** | 0,3085 | 0,1999 | **0,0185** | 0,1885 | 0,6359 | 0,8857 | 0,9788 |
| **batch #2 vs. batch #5** | 0,9724 | 0,3163 | 0,8705 | 0,8405 | **<0,0001** | 0,4918 | 0,2713 | 0,2504 | 0,3527 | 0,7283 | 0,9025 | 0,9894 |
| **batch #2 vs. batch #6** | 0,058 | **0,0013** | 0,2538 | 0,1277 | **0,0006** | 0,7795 | 0,9604 | 0,7458 | 0,9508 | 0,9993 | 0,99 | 0,9998 |
| **batch #3 vs. batch #4** | **0,0031** | 0,1452 | >0,9999 | 0,9051 | 0,7458 | 0,1523 | 0,0591 | **0,0021** | **0,0162** | **0,0049** | 0,7239 | 0,9077 |
| **batch #3 vs. batch #5** | 0,9961 | 0,9999 | 0,8886 | 0,9397 | 0,5975 | 0,2785 | 0,088 | 0,0548 | **0,0427** | **0,0078** | 0,7501 | 0,9397 |
| **batch #3 vs. batch #6** | 0,5975 | 0,4589 | 0,2749 | 0,221 | 0,9177 | 0,5493 | 0,7415 | 0,3203 | 0,4313 | 0,4496 | 0,9377 | 0,9925 |
| **batch #4 vs. batch #5** | **0,0146** | 0,237 | 0,8943 | >0,9999 | 0,9999 | 0,9996 | >0,9999 | 0,8827 | 0,9993 | >0,9999 | >0,9999 | >0,9999 |
| **batch #4 vs. batch #6** | **<0,0001** | **0,0007** | 0,2822 | 0,8187 | 0,999 | 0,9712 | 0,6737 | 0,3955 | 0,683 | 0,4132 | 0,9969 | 0,9975 |
| **batch #5 vs. batch #6** | 0,2971 | 0,3163 | 0,8914 | 0,7587 | 0,99 | 0,9973 | 0,7713 | 0,9604 | 0,8705 | 0,506 | 0,998 | 0,9993 |

**Supplementary Table 1 – Two way ANOVA multiple comparison analysis (p-values) of ELISA readouts for 2G4 serial dilutions of batch #1-6.** Duplicates per analysis were measured. Each cell mean was compared to every other cell mean of that row. In bold = p≤0.05


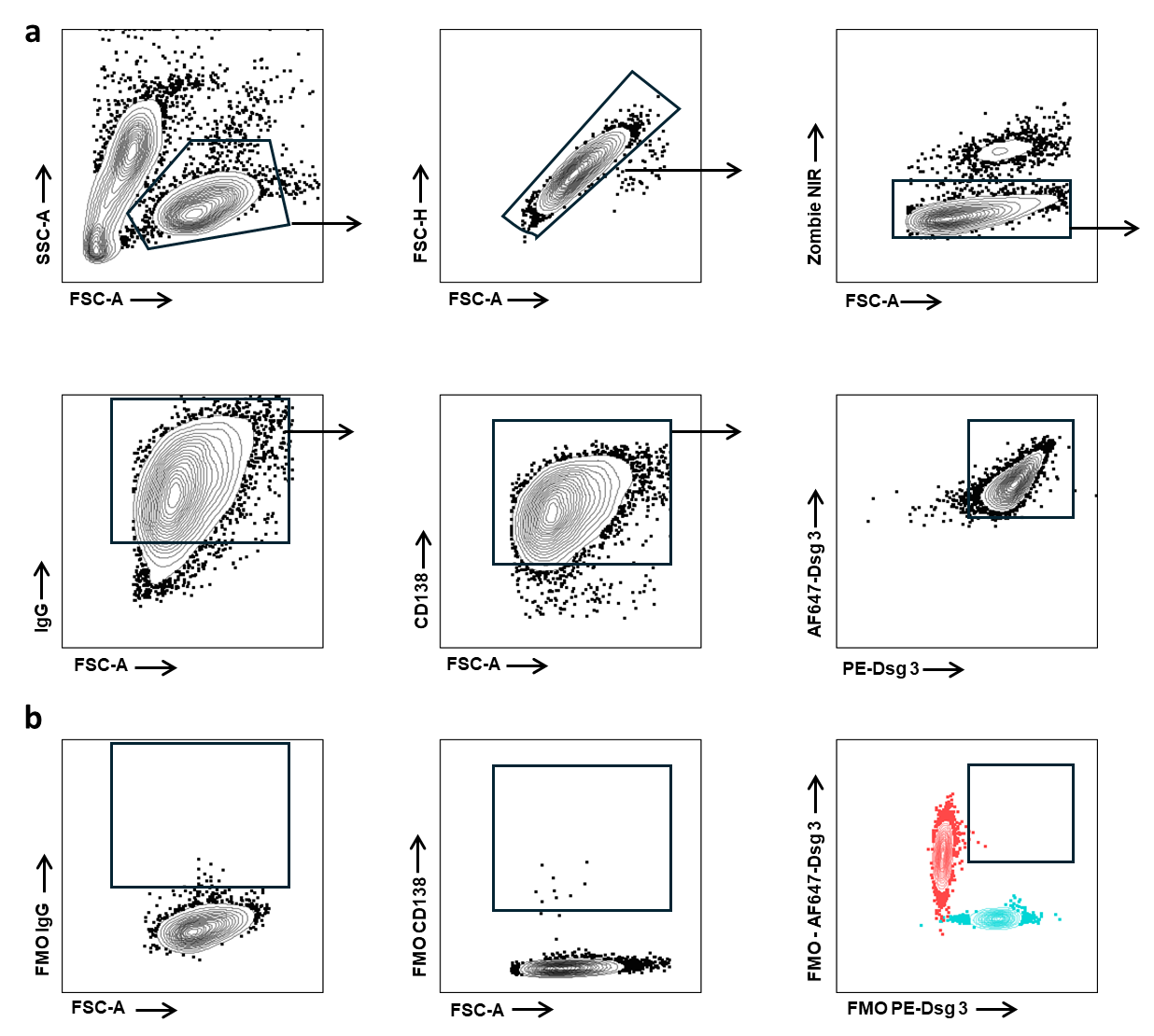


**Supplementary Figure 1 – Representative gating strategy for flow cytometric Dsg3-specific B cell identification.** Gating strategy identifying singlet and life cells, further characterized as IgG+ CD138+ followed by double positive staining for both AF647-Dsg3 and PE-Dsg3 **(a)**. Fluorescent-minus-one (FMO) display of used markers to verify staining procedure **(b)**.
